# Bilateral parasternal and rectus sheath blocks reduce pain post-cardiac surgery: a pilot trial

**DOI:** 10.3389/fsurg.2025.1526890

**Published:** 2025-02-20

**Authors:** Yangsi Huang, Chengdi Ouyang, Fang He, Yu Zhong, Guofeng Liu, Yizhi Lu, Yanhua Chen

**Affiliations:** Department of Anesthesiology, The First Affiliated Hospital of Guangxi Medical University, Nanning, Guangxi, China

**Keywords:** median sternotomy, parasternal block, postoperative analgesia, rectus sheath block, ropivacaine

## Abstract

**Objective:**

This study aimed to investigate the effects of ultrasound-guided bilateral parasternal block (PSB) combined with rectus sheath block (RSB) on postoperative recovery quality in patients undergoing median sternotomy for cardiac surgery.

**Methods:**

Eighty patients were randomly assigned to either the intervention group (receiving PSB + RSB, *n* = 40) or the control group (not receiving PSB + RSB, *n* = 40). The primary outcome was opioid consumption within the first 24 h postoperatively. Secondary outcomes included Visual Analog Scale (VAS) pain scores and various surgery and recovery-related parameters.

**Results:**

The intervention group showed significantly reduced opioid consumption in the first 24 h postoperatively compared to the control group (*P* < 0.05), though no significant difference was observed at 48 h postoperatively. VAS pain scores at extubation and at 12, 24, and 48 h post-extubation were significantly lower in the intervention group (*P* < 0.05). The intervention group also demonstrated superior Quality of Recovery-15 (QoR-15) scores at all observed time points compared to the control group (*P* < 0.05), with no block-related adverse events. There were no significant differences in surgical and recovery-related parameters between the groups.

**Conclusion:**

Ultrasound-guided bilateral PSB combined with RSB effectively enhances postoperative analgesia and the quality of recovery in patients undergoing median sternotomy for cardiac surgery. The application of ultrasound-guided bilateral parasternal block combined with rectus sheath block in median sternotomy cardiac surgery offers a new pain management strategy that is both safe and highly effective. This approach reduces postoperative analgesic requirements and improves recovery quality for cardiac surgery patients.

**Clinical Trial Registration:**

https://www.chictr.org.cn/showproj.html?proj=180456, China Clinical Trial Registry (ChiCTR2200064733).

## 1 Introduction

Traditional median sternotomy remains the primary approach for most cardiac surgeries. However, it is associated with significant trauma due to long incisions and sternal retraction, often resulting in severe postoperative pain. This pain can impede respiratory function recovery, early mobility, and overall quality of life (1, 2).

Modern pain management strategies emphasize multimodal analgesia, which combines various drugs and techniques to maximize pain relief while minimizing side effects (3, 4). Regional nerve blocks are a crucial component of multimodal analgesia (5, 6), as they enhance analgesic effects while reducing the need for systemic opioids by precisely blocking pain signal transduction pathways (5).

The parasternal block (PSB) is notable for its ease of use, safety, and effectiveness (7–9). Clinical trials have shown its efficacy in managing postoperative pain following median sternotomy (9–11). PSB effectively blocks the anterior cutaneous branches of the T2–T6 intercostal nerves, leading to improved pain control and better recovery quality post-cardiac surgery (10). While PSB offers significant benefits in controlling pain related to the anterior chest wall, it has its limitations. Its block range mainly focuses on the superficial nerves in the thoracic region and may not fully address the analgesic needs of cardiac surgery patients, especially when it comes to pain originating from deeper abdominal nerves and soft tissues (11).

The rectus sheath block (RSB) has been shown to provide effective trunk analgesia for midline incision (12). RSB involves the injection of anesthetic around the rectus sheath, targeting the abdominal wall's transverse nerves, such as the intercostal nerves from T7–T11 and the subcostal nerve (12–14). Its specific anatomical location and block range make it advantageous in controlling pain in the subxiphoid region, which is often affected by indwelling drainage tubes after cardiac surgery (9). In other words, RSB can target the areas that PSB might miss, complementing the analgesic effect. By blocking the nerves supplying the deeper abdominal structures and soft tissues, it fills the gap left by PSB. Therefore, combining PSB and RSB can theoretically offer more comprehensive and effective postoperative analgesia for cardiac surgery patients. Recent studies indicate that the combination of PSB and RSB significantly improves pain control at the thoracic drainage tube outlet in the upper abdominal region, reduces opioid use, and enhances respiratory function (13, 14).

Nevertheless, in the context of our study, when exploring the potential benefits of combining PSB and RSB, we were acutely aware of the need to account for possible confounding factors. In the initial stages of patient selection, we deliberately excluded those with a body mass index (BMI) greater than 28, as previous research has suggested that higher BMI could potentially influence the distribution and efficacy of nerve blocks, as well as postoperative pain perception. The variability in opioid use among patients could be influenced by multiple elements beyond just BMI. Patient characteristics such as age, which may affect the body's metabolism and pain tolerance, and pre-existing medical conditions like diabetes or cardiovascular diseases that could alter nerve function and the body's response to analgesia, might play a significant role in how effectively they respond to pain management strategies.

Assessing the quality of postoperative recovery is complex and requires consideration of both medical indicators and patients' personal experiences. The quality of recovery-15 (QoR-15) scale, comprising five dimensions and fifteen evaluation items, is a reliable tool for measuring postoperative recovery quality, with scores ranging from 0–150 (15). In this study, we conducted a pilot randomized, double-blind controlled trial to validate the effectiveness of combining PSB with RSB in reducing pain, decreasing opioid use, and improving the quality of recovery after cardiac surgery.

## 2 Methods

This study was conducted in accordance with the principles of the Declaration of Helsinki. The research took place from October 2022 to September 2025 and received approval from the Ethics Committee of the First Affiliated Hospital of Guangxi Medical University [Approval No. 2022-KY-(095)]. The trial was registered with the Chinese Clinical Trial Registry (ChiCTR2200064733). All participants provided written informed consent prior to enrollment.

### 2.1 Participants

Patients aged 18–65 years, who were scheduled for elective median sternotomy for cardiac surgery at the First Affiliated Hospital of Guangxi Medical University, and had an American Society of Anesthesiologists (ASA) physical status classification of I-III, were recruited for the study. Exclusion criteria included patients with severe cardiopulmonary, hepatic, or renal dysfunction; those with chest deformities preventing nerve block; individuals with a history of two or more previous thoracotomies; a body mass index (BMI) of 28 kg/m^2^ or higher; those with alcohol or opioid addiction; patients allergic to local anesthetics; those unwilling to participate; and patients with severe neurological or psychiatric disorders.

Patients were excluded if they experienced severe surgical or anesthetic complications in the perioperative period; were unable to cooperate with the visual analog scale (VAS) and QoR-15 scoring (16); required reoperation before discharge or after surgery; underwent operations lasting more than 7 h; did not undergo cardiopulmonary bypass; or experienced serious adverse reactions, significant physiological changes, or other unexpected events making it inappropriate to continue in the study.

### 2.2 Randomization and blinding

Patients were randomly assigned to either the control group or the intervention group using a computer-generated randomization table. The randomization sequence was created by an independent researcher who was not involved in the recruitment, treatment, or data analysis. Each participant was assigned a unique identifier, which was matched to the corresponding group allocation according to the randomization table. The allocation schedule was sealed in opaque envelopes, kept confidential, and securely stored. On the day before surgery, eligibility was confirmed, and written informed consent was obtained. On the day of surgery, an uninvolved individual opened the envelope to reveal the anesthetic plan. The randomization process and allocation concealment were periodically reviewed by an independent statistician to ensure strict adherence to the protocol. All other researchers and patients remained blinded to the allocation throughout the entire perioperative period.

### 2.3 Anesthetic preparation

Peripheral intravenous access was established, and continuous monitoring of electrocardiogram (ECG), pulse oximetry, and bispectral index (BIS) was implemented. Supplemental oxygen was delivered via a ventilator. Radial artery puncture and cannulation were performed under local infiltration with lidocaine, followed by the connection of an arterial sensor for continuous arterial blood pressure monitoring. Additionally, a right internal jugular vein puncture and cannulation were conducted under local infiltration with lidocaine for central venous pressure monitoring.

### 2.4 Anesthesia and postoperative management

Anesthesia was induced with an intravenous injection of penehyclidine hydrochloride 0.02 mg·kg^−1^, midazolam 0.1 mg·kg^−1^, etomidate 0.3 mg·kg^−1^, cisatracurium 0.2 mg·kg^−1^, and sufentanil 1 *μ*g·kg^−1^. After induction, tracheal intubation and mechanical ventilation were initated. Maintenance anesthesia involved continuous intravenous infusions of propofol 5–8 mg·kg^−1^·h^−1^, sufentanil 0.015 *μ*g·kg^−1^·min^−1^, and remimazolam tosylate 0.15 mg·kg^−1^·h^−1^. If mean arterial pressure (MAP) or heart rate (HR) increased during surgery, additional sufentanil sufentanil 0.2 *μ*g·kg^−1^ was administered, and intermittent doses of cisatracurium (0.1 mg·kg^−1^) were given to maintain muscle relaxation. BIS values were kept between 40 and 60 during the procedure. Potential adverse effects of anesthetic drugs, such as hypotension, bradycardia, or respiratory depression, were continuously monitored and promptly managed according to standard ICU protocols.

#### 2.4.1 Control group

Post-surgery, the control group was transferred directly to the cardiac surgery intensive care unit (ICU) while still intubated and monitored. Management followed the standard ICU protocol, including postoperative pain and respiratory management. Pain management involved continuous intravenous infusion of nalbuphine or remifentanil. The consumption of opioids was recorded to evaluate the total opioid dosage used in the postoperative period. Measures to mitigate opioid-induced adverse effects, such as nausea, vomiting, or hyperalgesia, included prophylactic administration of antiemetics and reducing opioid doses if hyperalgesia was suspected. After extubation, patients were transferred to the general cardiac surgery ward, where oral oxycodone was administered at regular intervals for analgesia. Visual analog scale (VAS) scores were recorded at extubation and at 12, 24, and 48 h post-extubation by personnel not involved in the trial.

#### 2.4.2 Intervention group

Upon surgery completion, the intervention group received an ultrasound-guided PSB combined with RSB using a total of 40 ml of 0.375% ropivacaine solution, with 20 ml administered on each side (total dose: 150 mg). The procedure started with the disinfection and draping of the chest area around the sternum and subxiphoid region. A high-frequency linear ultrasound probe, covered with a sterile sleeve, was used for guidance.

**Figure 1:**
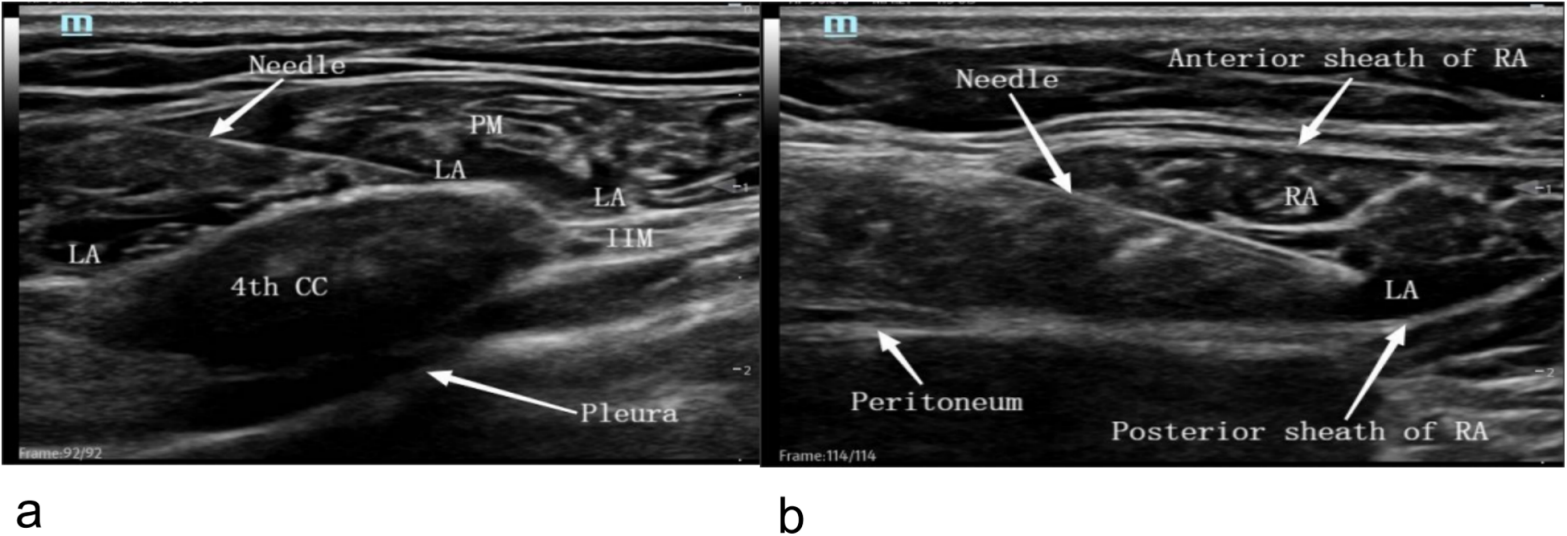
Ultrasound-guided block image. **(a)** parasternal block, **(b)** rectus abdominis sheath block. 4th CC, fourth costal cartilage; IIM, intercostal muscle; LA, local anesthetic, Needle, block needle; PM, pectoralis major; Pleura, pleura. Anterior sheath of RA, anterior sheath of rectus abdominis; LA, local anesthetic; Needle, blocking needle; Peritoneum, peritoneum; Posterior sheath of RA, posterior sheath of rectus abdominis; RA, rectus abdominis.

The ultrasound probe was positioned approximately 2 cm lateral to the sternum in a sagittal plane, visualizing the pectoralis major muscle, intercostal muscles, costal cartilage, pleura, and lung. The costal cartilage from the second to the sixth rib was identified, and the probe was placed above the second, fourth, and sixth rib cartilage. A 22G, 4-inch needle was advanced from the caudal to cranial direction until it reached the surface of the costal cartilage. Five milliliters of 0.375% ropivacaine was injected at the second, fourth, and sixth rib cartilage surfaces, with adjustments made as necessary to ensure even distribution along the fascia plane between the transverse thoracic muscle and the intercostal muscle above and below the costal cartilage (Figure 1a).

Next, the ultrasound probe was placed transversely above the ipsilateral rectus abdominis, approximately 3 cm below the xiphoid process. The rectus abdominis anterior sheath, rectus abdominis, rectus abdominis posterior sheath, and peritoneum were visualized. The needle was inserted laterally into the ultrasound plane, and 5 ml of 0.375% ropivacaine was injected between the rectus abdominis and its posterior sheath (Figure 1b). Blood aspiration was performed before injection to avoid intravascular administration or vascular injury. Any adverse effects related to local anesthetics, such as systemic toxicity, were actively monitored during and after block administration. This included continuous ECG, blood pressure, and oxygen saturation monitoring, as well as immediate readiness to administer lipid emulsion therapy if systemic toxicity occurred. All blocks were administered by a senior anesthesiologist experienced in ultrasound-guided nerve blocks.

After completing the nerve blocks, the patients were transferred to the cardiac surgery ICU with monitoring and endotracheal tubes in place. Postoperative management was consistent with the protocol followed for the control group.

### 2.5 Outcomes

#### 2.5.1 Primary outcome

The primary outcome was the opioids consumption within 24 h after surgery. measured in morphine milligram equivalents (MMEs).

#### 2.5.2 Secondary outcomes

Secondary outcomes included:

(1) Cumulative opioid consumption within 48 h post-surgery.
(2) VAS scores at extubation and at 12, 24, and 48 h post-extubation.
(3) QoR-15 scores 24 h before surgery and at 24, 48, and 72 h post-extubation.
(4) Operation time, transfer time, intraoperative sufentanil consumption, incidence of nausea and vomiting within 48 h post-operation, postoperative tracheal extubation time, ICU retention time, and hospitalization time.
(5) Adverse events related to intraoperative nerve blocks, such as puncture site hematoma, arteriovenous injury, ropivacaine allergy, pneumothorax, and puncture site infection.

### 2.6 Sample size and statistical analysis

Published study (17) indicate that patients consume an average of 31.4 ± 11.2 mg of morphine in the first 24 h post-surgery without a nerve block. To demonstrate a 25% reduction in postoperative morphine consumption (an absolute reduction of 7.9 mg) in the nerve block group, we calculated that 32 patients per group would be needed to achieve 80% power at a two-sided significance level of 0.05. The 25% reduction was chosen as a clinically significant effect size based on its relevance in previous pain management studies, which suggest that such a reduction is associated with meaningful improvements in patient outcomes, such as reduced side effects and shorter recovery times. To account for potential dropouts, we included a total of 80 patients.

Data were analyzed using SPSS 26 software. Normality was tested with the Shapiro–Wilk test. Normally distributed data were expressed as mean ± standard deviation. Repeated measures ANOVA was used to compare VAS scores between the two groups. Independent sample *t*-tests were used for comparisons of other normally distributed variables. Non-normally distributed data were expressed as median [interquartile range] and analyzed using the Scheirer-Ray-Hare test, which was selected due to its ability to account for both between-group and within-group effects in factorial designs with non-parametric data. This method was deemed more appropriate than the Kruskal–Wallis test, which is limited to single-factor designs. Group comparisons were conducted with the Mann–Whitney *U* test. Categorical variables were expressed as frequency and percentage, and compared using the chi-square test or Fisher's exact test. A *p*-value < 0.05 was considered statistically significant.

## 3 Results

**Figure 2:**
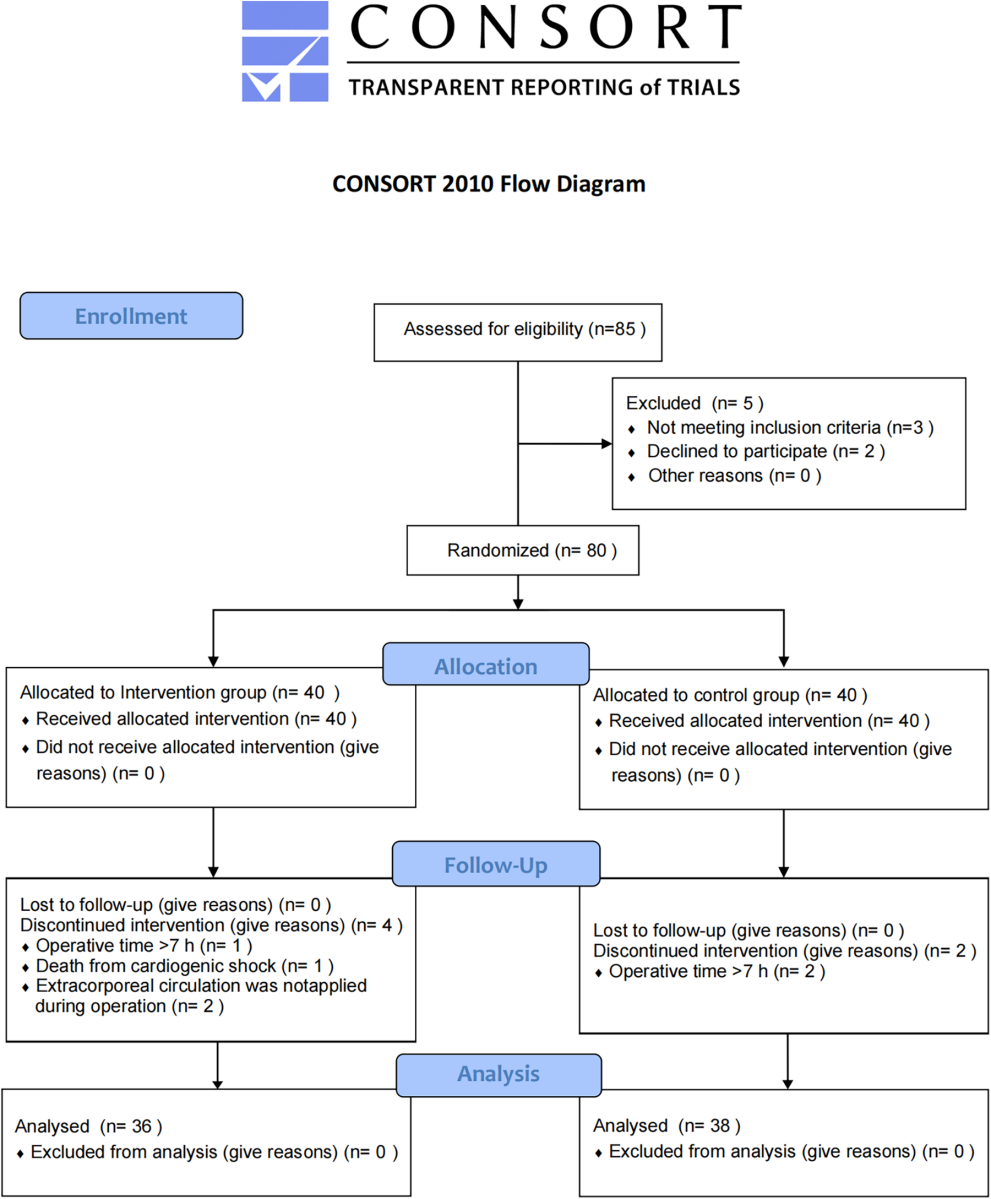
CONSORT flow diagram.

According to the CONSORT guidelines, the experimental flow chart for this study is shown in Figure 2. Eighty patients undergoing cardiac surgery with median sternotomy were initially included, with 40 patients in each group. Six patients were lost to follow-up: three due to operation time >7 h (one in the intervention group and two in the control group), one due to intraoperative cardiogenic shock (intervention group), and two because cardiopulmonary bypass was not used (intervention group). Consequently, 36 patients in the intervention group and 38 in the control group were included in the final analysis. The two groups were similar in terms of sex, age, BMI, and ASA classification (Table 1).

**Table 1:** Baseline characteristics of patients between groups.

Characteristic	Control (*n* = 38)	Intervention (*n* = 36)	*P*
Male, *n* (%)	19 (50.0%)	18 (50.0%)	1.00
Age, yr	48.4 ± 8.9	49.1 ± 10.9	0.84
BMI, kg·m^−2^	21.6 ± 2.9	22.6 ± 2.3	0.32
ASA, *n* (%)			1.00
II	12 (31.6%)	12 (33.3%)	
III	26 (68.4%)	24 (66.7%)	

The values are expressed as *n* (%) or mean ± standard deviation; ASA, American Society of Anesthesiologists; BMI, Body mass index.

### 3.1 Primary outcome

All subjects in the intervention group successfully underwent the PSB combined with RSB block. There were no complications such as hematoma at the puncture site, arteriovenous injury, ropivacaine allergy, local anesthetic toxicity, pneumothorax, or puncture site infection related to the nerve block. The cumulative consumption of MMEs in the intervention group was significantly lower within the first 12 h post-operation (32.7 ± 11.2 vs. 42.0 ± 9.8, *P* = 0.01).

There was no significant difference between the two groups in cumulative MME consumption within 48 h after surgery and in MME consumption between 24 and 48 h post-surgery, as shown in Table 2.

**Table 2:** Consumption of opioids between groups.

Outcome	Control(*n* = 38)	Intervention(*n* = 36)	*P*
Cumulative MMEs consumption			
Cumulative MMEs H0–H24, mg	42.0 ± 9.8	32.7 ± 11.2	0.01
Cumulative MMEs H0–H48, mg	69.0 ± 20.1	60.8 ± 23.9	0.28
Cumulative MMEs H24–H48, mg	27.0 ± 13.3	28.1 ± 15.1	0.82

The values are expressed as mean ± standard deviation. H0, patients with tracheal extubation; MMEs, morphine milligram equivalent.

### 3.2 Secondary outcomes

**Figure 3:**
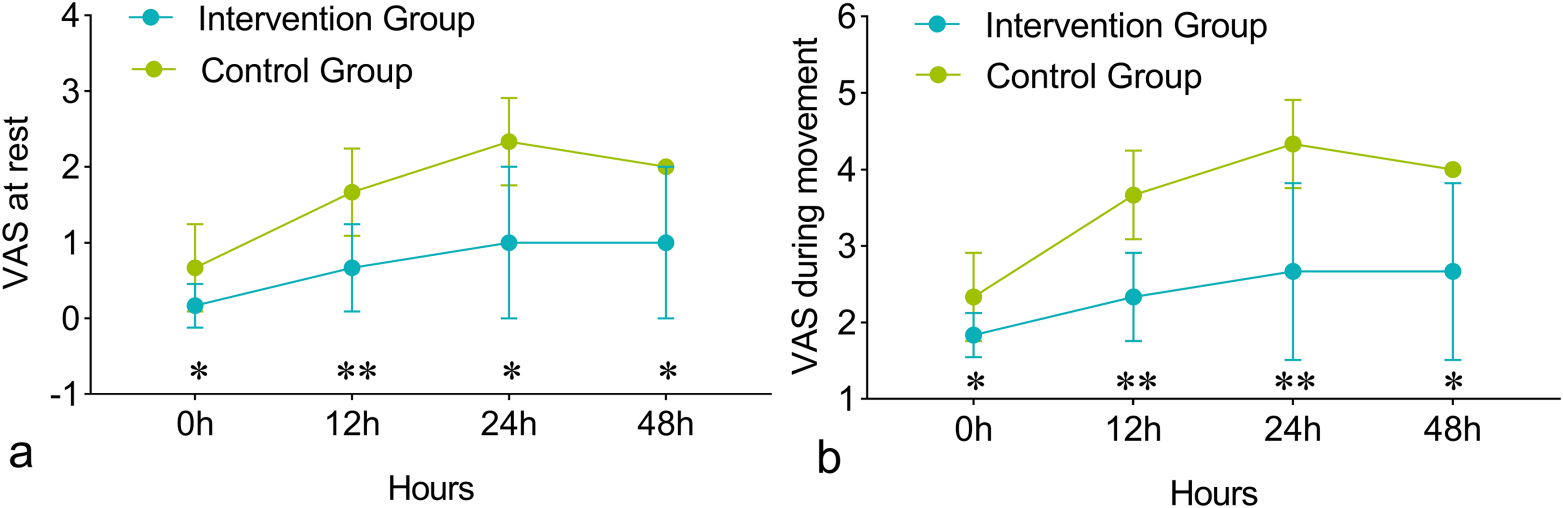
Comparison of visual analogue scale (VAS) pain scores between groups. VAS scores at rest **(a)** and during exercise **(b)** at 0, 12, 24, and 48 h post-extubation. The Scheirer-Ray-Hare test showed significant differences in group effect and time effect, with no significant interaction between group and time on VAS. *P < 0.05, **P < 0.01.

The Scheirer-Ray-Hare test analysis indicated that both group effect and time effect had significant impacts on pain scores at rest and during movement, while the interaction between group and time did not significantly affect pain scores. Compared with the control group, the intervention group had significantly lower pain scores at rest and during movement (Figure 3). The VAS scores at rest in the intervention group and the control group at extubation and 12, 24, and 48 h post-extubation were 0.00 (0.00, 1.00) VS 1.00 (0.00, 2.00) (*P* > 0.05), 1.00 (0.00, 1.00) VS 2.00 (1.00, 2.75) (*P* > 0.01), 1.00 (0.00, 2.00) VS 2.00 (2.00, 3.00) (*P* > 0.05), and 1.00 (0.00, 2.00) VS 2.00 (2.00, 3.00) (*P* > 0.05), respectively. The VAS scores during movement in the intervention group and the control group at extubation and 12, 24, and 48 h post-extubation were 2.00 (1.00, 2.00) VS 2.50 (2.00, 3.75) (*P* > 0.05), 2.00 (2.00, 3.00) VS 4.00 (3.00, 4.75) (*P* > 0.01), 2.00 (2.00, 4.00) VS 4.00 (4.00, 5.00) (*P* > 0.01), and 2.00 (2.00, 4.00) VS 4.00 (4.00, 5.00), respectively.

**Figure 4:**
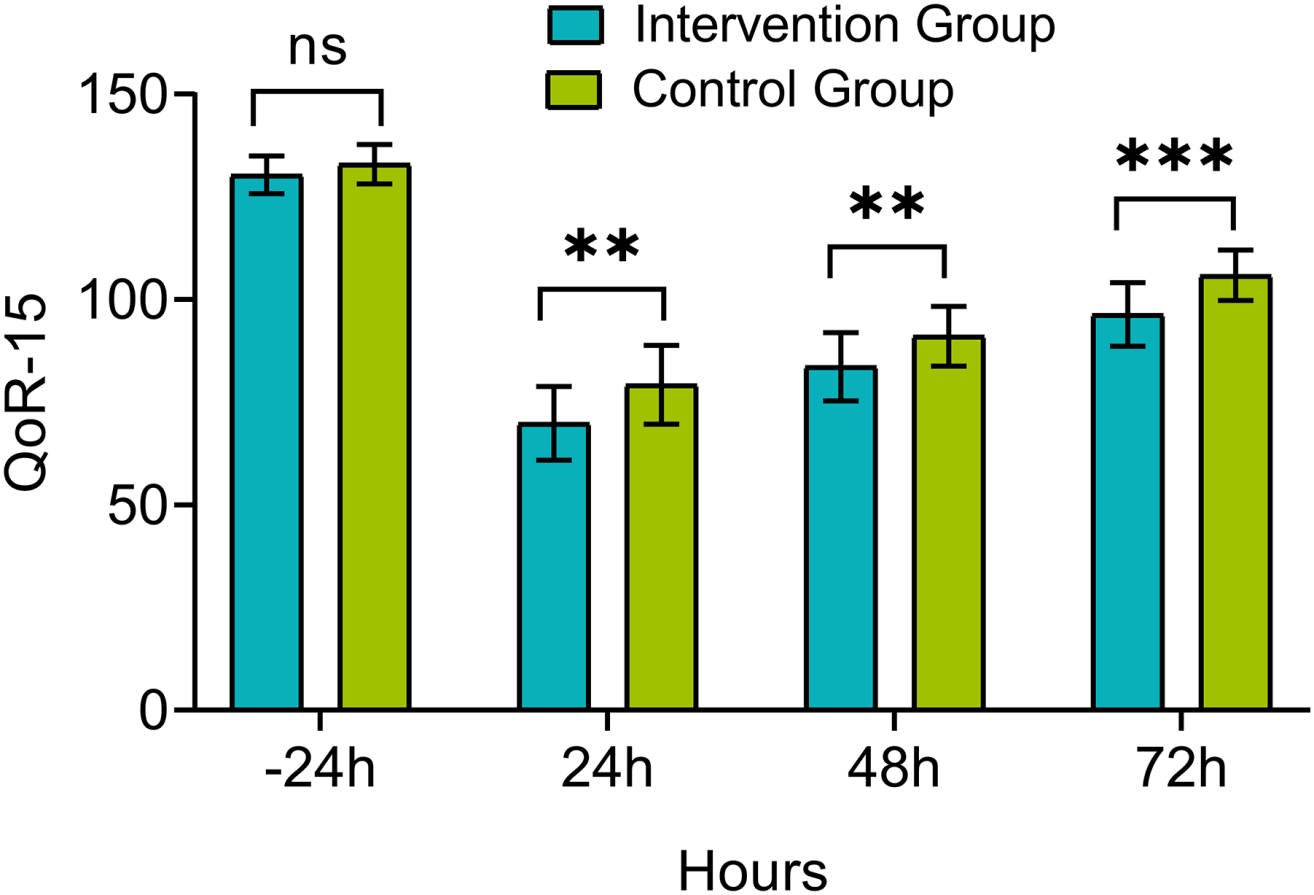
Comparison of quality of recovery-15 (QoR-15) scores between groups. Measurements were taken 24 h before extubation (−24 h) and at 24, 48, and 72 h post-extubation. ns, *P* > 0.05, *P < 0.05, **P < 0.01, ***P < 0.001.

There was no significant difference in QoR-15 score between the intervention group and control group 24 h before surgery (*P* > 0.05). However, the QoR-15 scores at 24, 48, and 72 h post-extubation were significantly higher in the intervention group compared to the control group (*P* < 0.05), as shown in Figure 4.

There was no significant difference between the two groups in operation time, transfer time, intraoperative sufentanil consumption, incidence of postoperative nausea and vomiting, postoperative tracheal extubation time, ICU retention time, or hospitalization time (Table 3, *P* > 0.05).

**Table 3:** Secondary outcomes between groups.

Outcome	Control(*n* = 38)	Intervention(*n* = 36)	*P*
Surgical duration, min	339 ± 57	298 ± 65	0.06
Bypass time, min	143 ± 40	120 ± 29	0.06
Intraoperative sufentanil, *μ*g	209 ± 28	205 ± 21	0.93
Nausea and vomiting, *n* (%)	9 (50%)	3 (18.8%)	0.08
Extubation time, h	20 ± 13.29	21 ± 16.41	0.96
ICU stay time, h	38 ± 24.59	46 ± 30.2	0.48
Hospitalization duration, d	15.3 ± 2.6	16.2 ± 4.9	0.47

*Note*: The values are expressed as *n* (%) or mean ± standard deviation. ICU, Intensive care unit.

## 4 Discussion

This study found that ultrasound-guided bilateral PSB combined with RSB block improves postoperative analgesia in patients undergoing median sternotomy cardiac surgery. Compared with no PSB combined with RSB, the combined block reduced opioid demand, with a 22% (9.3 mg) decrease in MME consumption within the first 24 h post-surgery. Additionally, bilateral PSB combined with RSB significantly reduced VAS pain scores and improved QoR-15 scores. There were no significant differences in operation time, transfer time, intraoperative sufentanil consumption, postoperative nausea and vomiting, postoperative tracheal extubation time, ICU retention time, or hospital stay between the groups.

In cardiac surgery, epidural analgesia poses certain risks due to heparinization treatment and the potential hemodynamic fluctuations caused by the surgery itself. However, apart from epidural analgesia, other regional anesthetic techniques like paravertebral space block, quadratus lumborum block, and erector spinae plane block also play significant roles in pain management for patients undergoing elective sternotomy. Each of these techniques has its own unique features and considerations. For instance, paravertebral space block provides segmental anesthesia and can offer effective pain relief with relatively fewer systemic side effects compared to some other methods. Quadratus lumborum block targets the muscles in the lumbar region and may have particular advantages in certain patient populations. Erector spinae plane block, on the other hand, has shown promise in providing analgesia for thoracic surgeries. As an alternative, PSB offers comparable pain control to epidural analgesia during sternotomy while avoiding the complications associated with epidural analgesia (18). This local anesthesia technique reduces the risk of bleeding and avoids issues related to epidural catheters, providing a safe and effective option for postoperative pain management in cardiac surgery. However, there are limitations to the application of PSB for postoperative pain relief in cardiac surgery. This pain not only includes the surgical incision but also the upper abdominal discomfort caused by the friction between the drainage tube and the muscle (19). Although traditional PSB effectively alleviates chest incision pain, its impact on upper abdominal pain is limited. To address this issue, we introduced RSB, which can reduce upper abdominal pain, especially that caused by the drainage tube (20).

Our results showed that within 24 h after cardiac surgery, patients receiving PSB combined with RSB exhibited a significant reduction in opioid consumption compared to the control group. However, this significance was not maintained at subsequent time points after surgery. In contrast, Wang et al. (14) found that in cardiac surgery, opioid consumption in patients receiving pectoral interfacial plane block (PIFB) combined with RSB was reduced by 38.8% and 35.0% within 24 and 48 h postoperatively, respectively, compared to patients receiving PIFB alone. It is worth noting that Piraccini et al. indicated that PIFB and PSB are essentially different names for the same block technique, both involving local anesthetic injections between the pectoralis major and intercostal muscles to achieve analgesia (21). The lesser reduction in opioid consumption observed with PSB combined with RSB in our study may be attributed to the use of dexamethasone in Wang et al.'s study. Dexamethasone, used as an adjunct to local anesthetic ropivacaine for PIFB, can prolong the duration of the block and reduce the production of inflammatory factors (22, 23). Additionally, the longest analgesic duration of ropivacaine for fascia block is about 9 h (24), which might explain why a continuous significant difference in opioid consumption was not observed within 48 h postoperatively in our study. This phenomenon may also be related to the differences in pain perception among individual patients, variations in the metabolic pathways of opioid drugs, and the rapid metabolism of ropivacaine. In particular, the postoperative inflammatory response may be further alleviated within 48 h, reducing the need for additional analgesia (25). Despite the modest reduction in opioid consumption with PSB combined with RSB, its advantages were still notable compared to the control group without nerve block.

Contrastingly, Pascarella et al.'s study showed no significant reduction in opioid use within 24 h after ultrasound-guided PSB (10). We believe this discrepancy may be due to the absence of RSB in their nerve block regimen. As a complementary block, RSB has proven effective in controlling specific pain areas after cardiac surgery, especially drainage-related pain. Cardiac surgery pain is multifaceted, involving incision, sternal traction, musculoskeletal injury, and drainage tube insertion points (25). Therefore, a single PSB may not sufficiently cover all pain sources. Our study's results align with those of Strumia et al. and Wang et al. (13, 14), indicating that PSB combined with RSB has advantages in postoperative analgesia. On one hand, pain sources in cardiac surgery are multifaceted, encompassing the surgical incision, sternal traction, musculoskeletal injury, and drainage tube insertion sites. Compared to single nerve block techniques that address only partial pain sources, the combination of PSB and RSB can block the T2–T8 anterior cutaneous branches, covering the sternal incision, subxiphoid incision, and subxiphoid drainage tube areas, thereby expanding the analgesic scope. On the other hand, traditional PSB is limited in addressing upper abdominal discomfort caused by friction between the drainage tube and muscle, a unique pain challenge in cardiac surgery. As a complementary technique, RSB effectively controls this pain. Together, the synergy of PSB and RSB provides a comprehensive solution to postoperative complex pain, offering a novel approach to optimizing pain management strategies.

VAS is a widely used tool for assessing postoperative pain management in cardiac surgery, with a minimum clinically important difference of 1.0 (26). In this study, the combination of PSB and RSB significantly reduced the VAS score both at rest and during activity after extubation, surpassing this threshold and indicating a notable improvement in pain control. Additionally, we evaluated the quality of recovery within the first three days post-surgery. The QoR-15 scores at 24 and 72 h postoperatively were significantly lower, likely due to optimized pain control. Although the difference between the groups at 48 h did not meet the minimum clinically important difference value of 8 (27), when considering the reduction in VAS score and opioid consumption, we believe that PSB combined with RSB provides effective analgesia during the perioperative period.

A combination of continuous PIFB and RSB, covering T1–T10, has been effectively utilized to provide substantial analgesia in cardiac surgery (28). Our study further supports this approach, demonstrating that this combined block offers more extensive and comprehensive analgesia for patients undergoing median thoracotomy open heart surgery. Furthermore, although the improvement in the QoR-15 score at 48 h post-surgery did not fully reach the minimum clinically important difference, even small changes in scores can have a practical impact on patients' daily activities and overall satisfaction. When evaluating the effect of clinical interventions, it is essential to consider not only statistical significance but also the actual impact on patients' quality of life. We acknowledge that the observed changes in QoR-15 scores, although modest, may reflect meaningful improvements in areas such as pain management, emotional well-being, and physical recovery. These domains are crucial for patients' daily functioning and longer-term rehabilitation outcomes. Future studies focusing on the integration of these findings with patient-centered outcomes, including health-related quality of life and functional recovery metrics, would provide a more comprehensive understanding of the clinical significance of these interventions. Therefore, we consider this finding to be of significant clinical relevance.

In terms of safety, no serious complications were observed in this trial. However, underlying heart disease can be crucial to postoperative recovery. While PSB combined with RSB may be beneficial for pain management, other factors during surgery and recovery, such as operation time, medication usage, postoperative complications, and individual patient differences, should also be considered when evaluating pain management protocols.

This study had several limitations. Firstly, the sample size was relatively small. Although this study achieved the preset statistical power of 80%, as a single-center study, the external validity of its results still needs to be carefully considered. The strict inclusion and exclusion criteria of the study (such as excluding patients with BMI ≥ 28 kg/m^2^ and severe cardiopulmonary insufficiency) significantly limit the generalization of the results to a broader patient population. Especially for patients with more comorbidities or a higher body mass index, the safety and effectiveness of this method still need further verification. Another important limitation was the timing of the nerve blocks, which were performed after surgery. Administering a pre-sternotomy block could have potentially reduced intraoperative and perioperative opioid consumption, enhancing overall pain management. Additionally, the use of single-shot blocks rather than continuous catheter techniques limited the duration of analgesia to less than 24 h. While continuous catheter techniques could ensure prolonged pain relief and reduce opioid use, they were not adopted in this study, as the primary focus was to evaluate the efficacy of single-shot blocks for short-term pain management. This design inherently restricts the scope of analgesic effects assessed. Furthermore, this study primarily evaluated the short-term outcomes within 72 h post-surgery. To address this limitation, future studies should consider a multi-center design with an extended follow-up period of at least 3 months to assess long-term outcomes, including chronic pain and functional recovery. Additionally, including a broader patient population will be essential to determine the generalizability and broader applicability of the combined block technique across diverse clinical settings.

By addressing these limitations and pursuing these future research directions, we can gain a deeper understanding of the effectiveness and safety of the combined PSB-RSB blockade in enhancing postoperative analgesia and recovery following cardiac surgery.

## 5 Conclusion

In summary, ultrasound-guided bilateral PSB combined with RBS appears to be a promising approach that shows potential in enhancing postoperative analgesia and improving recovery quality following median sternotomy cardiac surgery. While initial findings suggest its efficacy and safety, further research is warranted, especially regarding long-term outcomes and its safety in larger patient populations. These preliminary findings tentatively support the exploration of this multimodal analgesic approach in managing pain after cardiac surgery, potentially offering an alternative to traditional pain management strategies.

## Data Availability

The raw data supporting the conclusions of this article will be made available by the authors, without undue reservation.
